# Lymph node density as a prognostic predictor in patients with betel nut-related oral squamous cell carcinoma

**DOI:** 10.1007/s00784-017-2247-3

**Published:** 2017-10-17

**Authors:** Wei-Chin Chang, Chun-Shu Lin, Cheng-Yu Yang, Chih-Kung Lin, Yuan-Wu Chen

**Affiliations:** 1 Department of Oral and Maxillofacial Surgery, Tri-Service General Hospital, Taipei City, Taiwan Republic of China; 2 School of Dentistry, National Defense Medical Center, Taipei City, Taiwan Republic of China; 3 Department of Radiation Oncology, Tri-Service General Hospital, Taipei City, Taiwan Republic of China; 4 Department of Pathology, Taipei Tzu Chi Hospital, Buddhist Tzu Chi Medical Foundation, Taipei City, Taiwan Republic of China; 5 Division of Oral and Maxillofacial Surgery, Tri-Service General Hospital, 2F, No. 325, Sec. 2, Chenggong Rd., Neihu District, Taipei City, 114 Taiwan Republic of China

**Keywords:** Betel nut, Lymph node density, Oral squamous cell carcinoma, Prognostic factor

## Abstract

**Objectives:**

Lymph node metastasis in oral squamous cell carcinoma (OSCC) is a poor prognostic factor. The histopathologic stage (e.g., pN) is used to evaluate the severity of lymph node metastasis; however, the current staging system insufficiently predicts survival and recurrence. We investigated clinical outcomes and lymph node density (LND) in betel nut-chewing individuals.

**Material and methods:**

We retrospectively analyzed 389 betel nut-exposed patients with primary OSCC who underwent surgical resection in 2002–2015. The prognostic significance of LND was evaluated by overall survival (OS) and disease-free survival (DFS) using the Kaplan-Meier method.

**Results:**

Kaplan-Meier analyses showed that the 5-year OS and DFS rates in all patients were 60.9 and 48.9%, respectively. Multivariate analysis showed that variables independently prognostic for OS were aged population (hazard ratio [HR] = 1.6, 95% confidence interval [95% CI] = 1.1–2.5; *P* = .025), and cell differentiation classification (HR = 2.4, 95% CI = 1.4–4.2; *P* = .002). In pathologic N-positive patients, a receiver operating characteristic (ROC) curve for OS was used and indicated the best cutoff of 0.05, and the multivariate analysis showed that LND was an independent predictor of OS (HR = 2.2, 95% CI = 1.3–3.7; *P* = .004).

**Conclusions:**

Lymph node density, at a cutoff of 0.05, was an independent predictor of OS and DFS. OS and DFS underwent multiple analyses, and LND remained significant. The pathologic N stage had no influence in the OS analysis.

**Clinical relevance:**

LND is a more reliable predictor of survival in betel nut-chewing patients for further post operation adjuvant treatment, such as reoperation or adjuvant radiotherapy.

## Introduction

Oral squamous cell carcinoma (OSCC) is the sixth most prevalent malignancy worldwide and the most frequent malignant tumor of the oral cavity [1]. In Taiwan, the areca nut is often chewed with the betel leaf [2]; therefore, OSCC ranks the fourth most prevalent cancer in the Taiwanese male population and the sixth most prevalent cancer in both sexes [3]. Alkaloids and nitrosamines found in the betel nut are confirmed carcinogens that produce malignant or precancerous lesions [4]. Nearly 2.5 million people consume betel nuts, and higher rates of mortality and recurrence exist in Taiwan [3]. However, research regarding the prognosis of patients who chew betel nuts is limited, and the prognosis in these cases is also related to the adjuvant treatment.

Treatment selection follows the National Comprehensive Cancer Network (NCCN) guidelines [5], with surgery combined with adjuvant concurrent chemo and/or radiotherapy being the primary treatment for OSCC [6–8]. The therapeutic mode is based on the initial stage, the tumor, node, metastasis (i.e., TNM) staging system, and the final histopathologic adverse features. Histopathologic findings of pathologic N (pN) status and lymph node metastases have been associated with a poor outcome [9]. However, pN staging is not specifically compatible with overall survival, since it is affected by surgical technique, dissection amount, and pathologic scrutiny [10]. Therefore, an alternative staging system for the survival evaluation has emerged.

The definition of lymph node density (LND) is the number of positive nodes divided by total number of resected nodes. LND is an important factor for prognostic survival prediction, which was first confirmed in a survival analysis of patients with carcinoma of the bladder [11] and the esophagus [12]. Histopathologically positive lymph nodes were identified based on surgical lymph nodal dissection with the pathologist’s sampling procedure. The feasibility of distinguishing metastatic lymph nodes is influenced by the technical performance of surgeons and pathologists. LND has emerged as an independent prognostic factor and demonstrates fewer sensitivity errors in terms of sampling technique.

To date, no study has evaluated the utility of LND to predict prognosis in patients with OSCC who regularly chew betel nut. The optimal cutoff value of LND could be a practical prognostic predictor, and adjuvant treatment could be administered based on this LND value. Therefore, we aimed to compare surgical outcomes by LND, tumor differentiation, primary site, and TNM staging.

### Patients and methods

Patients with treated primary OSCC who underwent surgical resection from 2002 to 2015 were included in this study. A total of 389 patients with a history of betel nut exposure with and without a betel nut-chewing habit were analyzed. All patients chewed at least one quid of betel nuts, including any type of betel product, every day for a minimum half of the year. Having a betel nut-chewing habit was defined as consumption of at least 10 nuts per day for more than 5 years, preceding the first survey appointment. All patients with primary OSCC received a minimum of selective neck lymphatic node dissection. We excluded patients who were initially diagnosed as having distant metastases. Only primary OSCC patients with safe margins were included. Adjuvant radiotherapy or concurrent chemoradiotherapy was administered after staging and if adverse features were present. All patients were followed and registered in an institutional database updated with the patients’ latest treatment condition. The follow-up duration was at least 2 years or until death by an endpoint of December 2015. All patients provided informed consent and the Ethical Committee of the Tri-Service General Hospital (Taipei, Taiwan) approved this retrospective study (institutional review board protocol no: I-105-05-049).

The definitive tumor staging relied on pathological features, based on the American Joint Committee on Cancer staging. The definitive nodular staging was also based on the collected pathological specimen from the selective neck dissection. Patients with extracapsular nodal spread, which is a feature of poor outcome, were excluded from this study. We compared surgical outcome according to LND, tumor differentiation, primary sites, and TNM staging.

### Statistical analyses

The chi-square test was used to analyze categorical variables. The Kaplan-Meier method was used to calculate overall survival (OS) and disease-free survival (DFS). OS was measured from the day of therapeutic surgery to the date of death, or the last follow-up, and these dates were registered by the cancer-recorded group. The DFS was measured from the day of the surgery to the date of tumor recurrence, which was defined as either local or distant metastasis. The OS and DFS were measured using different categories of LND values. A receiver operating characteristic (ROC) curve for OS from the start of treatment was utilized to verify the optimal cutoff values for LND.

The tumor differentiation, stage, pathological tumor (pT) classification, and pN status were analyzed for comparisons using regression. The statistical analysis was conducted using Cox regression and the Kaplan-Meier method via SPSS statistical software v20.0 (IBM Corp., Armonk, NY). The Kaplan-Meier estimator was used to evaluate the survival rate with a given LND cutoff value. A value of *p* < 0.05 was considered statistically significant.

## Results

### Patient demographics

Patient demographics, which include age, sex population, histopathologic T stage, N stage, treatment modality, and anatomical site, are presented in Table 1. The final histopathologic reports showed 133 (34.2%) patients with lymph node-positive disease and 256 (65.8%) patients with a pN0 status. Based on evidence from the literature, alcohol, betel nut, and cigarette consumption can cause OSCC and related cancers [13]. Three-hundred and fourteen (80.7%) patients smoked and 292 (75.1%) patients had an alcohol drinking habit. All patients included in this study had a history of betel nut chewing; however, only 305 (78.4%) patients maintained a betel nut-chewing habit at the time of the first medical consultation.

**TABLE 1:** Demographics of the patients

Characteristics		No. of patients	Percentage (%)
Sex	Men	355	91.3
	Women	34	8.7
Mean age (year)	51.8 (range, 23–84)	389	100.0
Tobacco exposure	No	75	19.3
	Yes	314	80.7
Alcohol exposure	No	97	24.9
	Yes	292	75.1
Betel nut-chewing habit	No	84	21.6
	Yes	305	78.4
Overall TNM stage	I	99	25.4
	II	85	21.9
	III	64	16.5
	IV	141	36.2
T classification	1	119	30.6
	2	125	32.1
	3	43	11.1
	4	102	26.2
N classification	N0	256	65.8
	N1	55	14.1
	N2a	2	0.5
	N2b	64	16.5
	N2c	11	2.8
	3	1	0.3
Treatment	Surgery only	106	27.2
	Surgery + RT	69	17.7
	Surgery + CT	56	14.4
	Surgery + CCRT	158	40.6
Anatomical site	Lip	2	0.5
	Retromolar trigone	18	4.6
	Gingiva	52	13.4
	Tongue	170	43.7
	Palate	9	2.3
	Buccal mucosa	127	32.6
	Mouth floor	11	2.8
Follow-up duration for all patients (months)	Mean: 50.3 ± 35.8	389	100.0
	Median: 42		
	Range: 0–152		

*CCRT*, concurrent chemoradiotherapy; *CT*, chemotherapy; *RT*, radiotherapy; *TNM*, tumor, node, metastasis

**Fig. 1:**
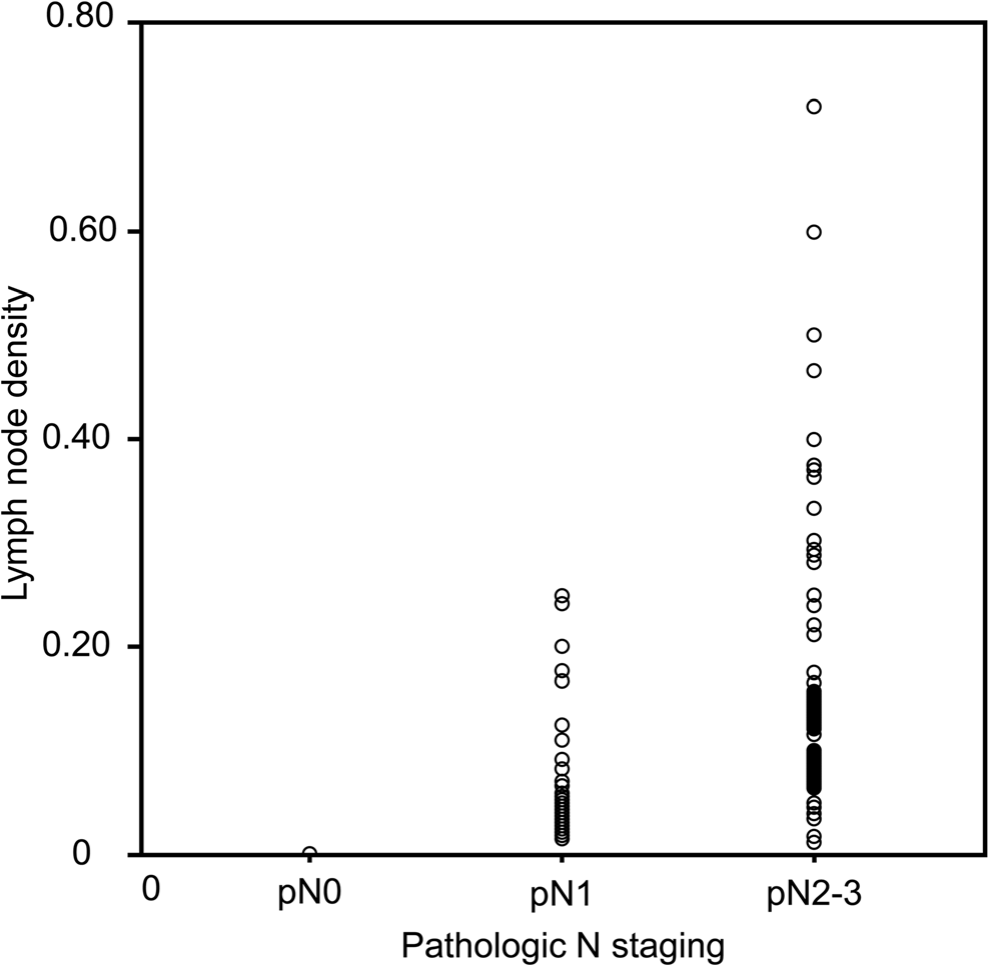
The lymph node density value is significantly different between the three histopathologic nodal stages (*p* < 0.001)

**Fig. 2:**
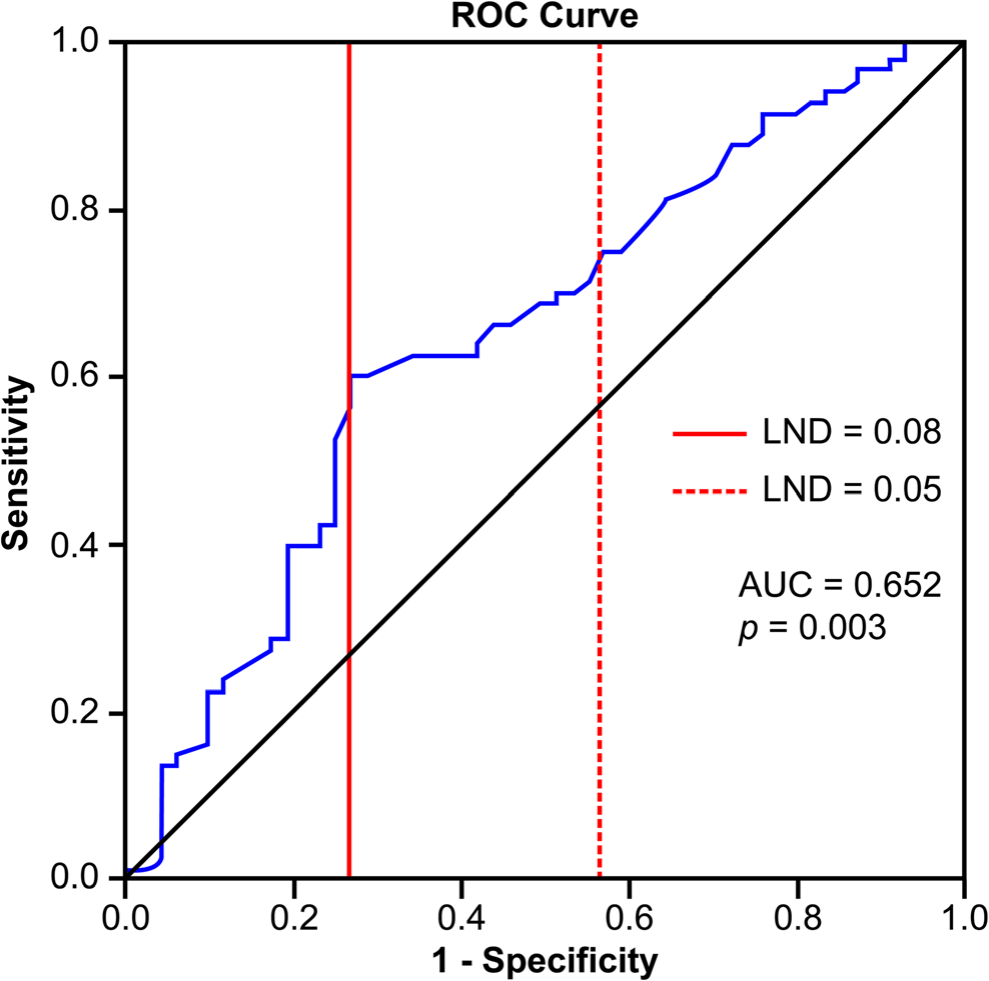
The receiver operating characteristic (ROC) curve for the LND value for overall survival of patients with pathologic-positive results. The red solid line indicates an LND value of 0.08 and the red dotted line indicates an LND value of 0.05. The Youden index (YI), for an LND value of 0.08 is 1.324 and for an LND value of 0.05, the YI is 1.184. The LND value of 0.05 is the optimal cutoff value since the sensitivity is much higher than for an LND of 0.08. (AUC, area under the curve)

The LND values among the patients with a pN stage are shown in Fig. 1. The mean LND was 0.043 ± 0.094 for all patients, and the mean LND was 0.126 ± 0.124 for the histopathologic-positive cases. The ROC curves for OS were plotted to verify the optimal cutoff values for a given LND values (Fig. 2). Based on the Youden index (YI) [14], the value of YI for the LND value of 0.08 is 1.324 and for the LND value of 0.05 is 1.184. The LND value of 0.05 is the best cutoff value due to a higher associated sensitivity than for an LND value of 0.08, with respective sensitivities of 78.0 and 62.5%.

Based on the histopathologic results (using a cutoff value of 0.05), the LND was categorized into three groups: 259 (66%) patients with a proven pN0 status (group A), 49 (13%) patients with an LND ≤ 0.05 (group B), and 84 (21%) patients with an LND > 0.05 (group C). The characteristics of these three groups were compared (Table 2). The distribution indicated significant differences in treatment, stage, alcoholism, pT status, and pN status.

**TABLE 2:** Comparison of the patients’ characteristics, based on group

Variable	Group A LND = 0 *n* = 256	Group B LND < 0.05 *n* = 49	Group C LND ≥ 0.05 *n* = 84	*p*
Age (year)				0.415
Median	52	50	52	
Sex				0.430
Men	231 (90.2)	47 (95.9)	77 (91.7)	
Women	25 (09.8)	2 (04.1)	7 (08.3)	
T classification				0.000*
T1 + T2	184 (71.9)	23 (46.9)	37 (44.0)	
T3 + T4	72 (28.1)	26 (53.1)	47 (56.0)	
N classification				0.000*
N0	256 (100.0)	0 (00.0)	0 (00.0)	
N1	0 (00.0)	37 (75.5)	18 (21.4)	
N2/N3	0 (00.0)	12 (24.5)	66 (78.6)	
Stage				0.000*
I	99 (38.7)	0 (00.0)	0(00.0)	
II	85 (33.2)	0 (00.0)	0 (00.0)	
III	23 (09.0)	28 (57.1)	13 (15.5)	
IV	49 (19.1)	21 (42.9)	71 (84.5)	
Treatment				0.000*
Surgery only	93 (36.3)	5 (10.2)	8 (09.5)	
Surgery + RT	51 (19.9)	7 (14.3)	11 (13.1)	
Surgery + CT	45 (17.6)	4 (08.2)	7 (08.3)	
Surgery + CCRT	67 (26.2)	33 (67.3)	58 (69.0)	
Tobacco exposure				0.752
Positive	204 (79.7)	40 (81.6)	70 (83.3)	
Negative	52 (20.3)	9 (18.4)	14 (16.7)	
Alcoholism				
Positive	182 (71.1)	38 (77.6)	72 (85.7)	0.025*
Negative	74 (28.9)	11 (22.4)	12 (14.3)	
Betel nut habit				0.270
Positive	195 (76.2)	42 (85.7)	68 (81.0)	
Negative	61 (23.8)	7 (14.3)	16 (19.0)	

Unless otherwise indicated, the data are presented as the number (%)

*LND*, lymph node density

*Indicates a significant difference, *p* < 0.05

### Survival analysis

**Fig. 3:**
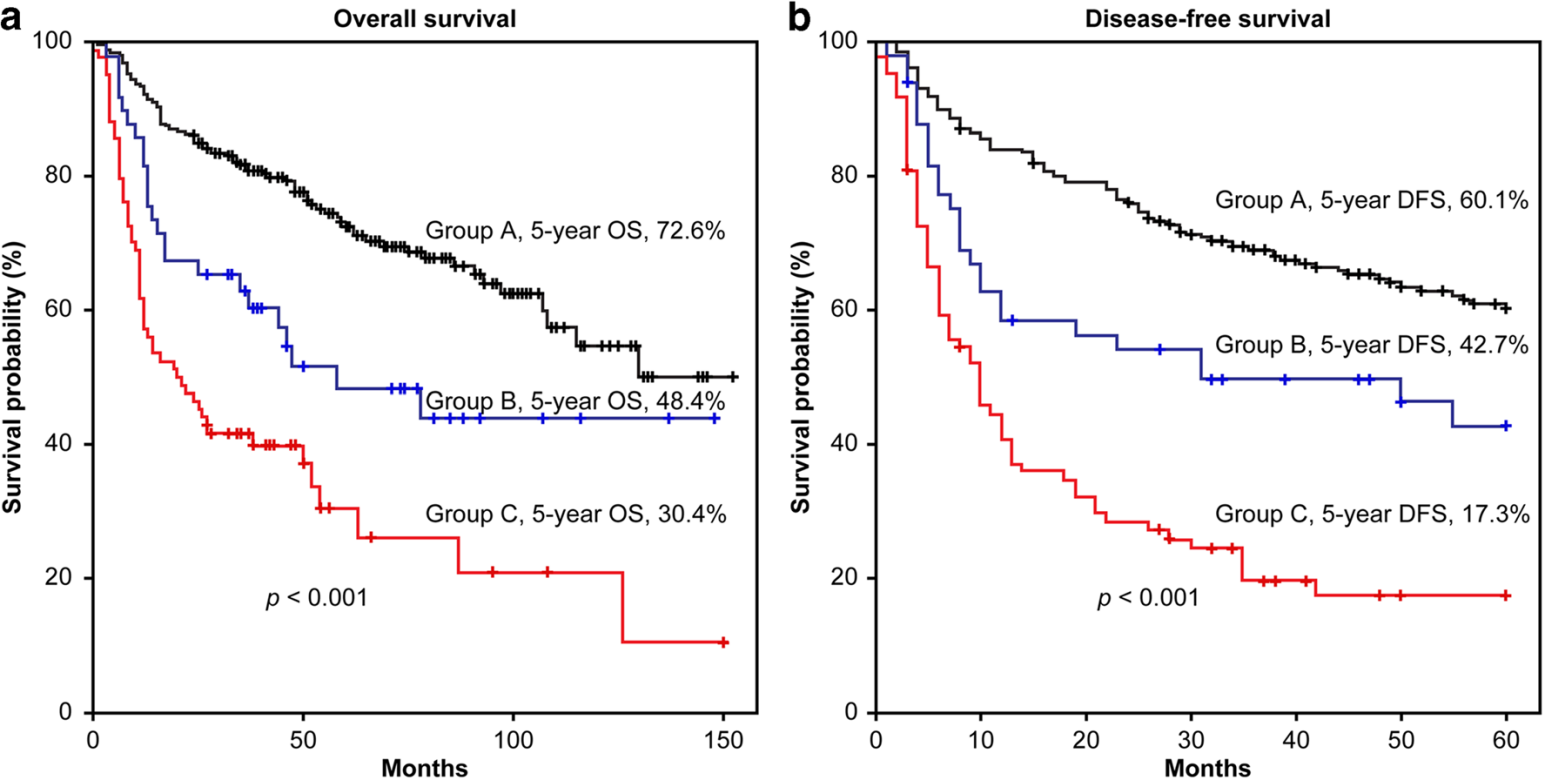
Kaplan-Meier plots for stratification by group of (**a**) 5-year survival and (**b**) 5-year disease-free survival. In group A, the LND is 0; in group B, the LND is 0 to 0.05; and in group C, the LND is ≥ 0.05

The mean (standard deviation [SD]) overall follow-up period was 50 (35) months among the 389 patients. The 1-year, 3-year, and 5-year OS were 83.3, 70.8, and 60.9%, using the Kaplan-Meier method. The 1-year, 3-year, and 5-year DFS were 71.5, 56.1, and 48.9%, respectively. The 5-year OS in group A, group B, and group C were 72.6, 48.4, and 30.4%, respectively (*p* < 0.001; Fig. 3a). The 5-year DFS in group A, group B, and group C were 60.1, 42.7, and 17.3%, respectively (*p* < 0.001; Fig. 3b). One-hundred fifty-six (40.1%) patients died due to cancer progression; the mean survival period was 27.4 months (SD = 27.6 months). One-hundred and eighty-five patients had locoregional recurrence or distant metastasis, and the mean disease survival period was 15.2 months (SD = 14.1 months). Among the three groups, LND value significantly predicted the 5-year OS and DFS.

**Fig. 4:**
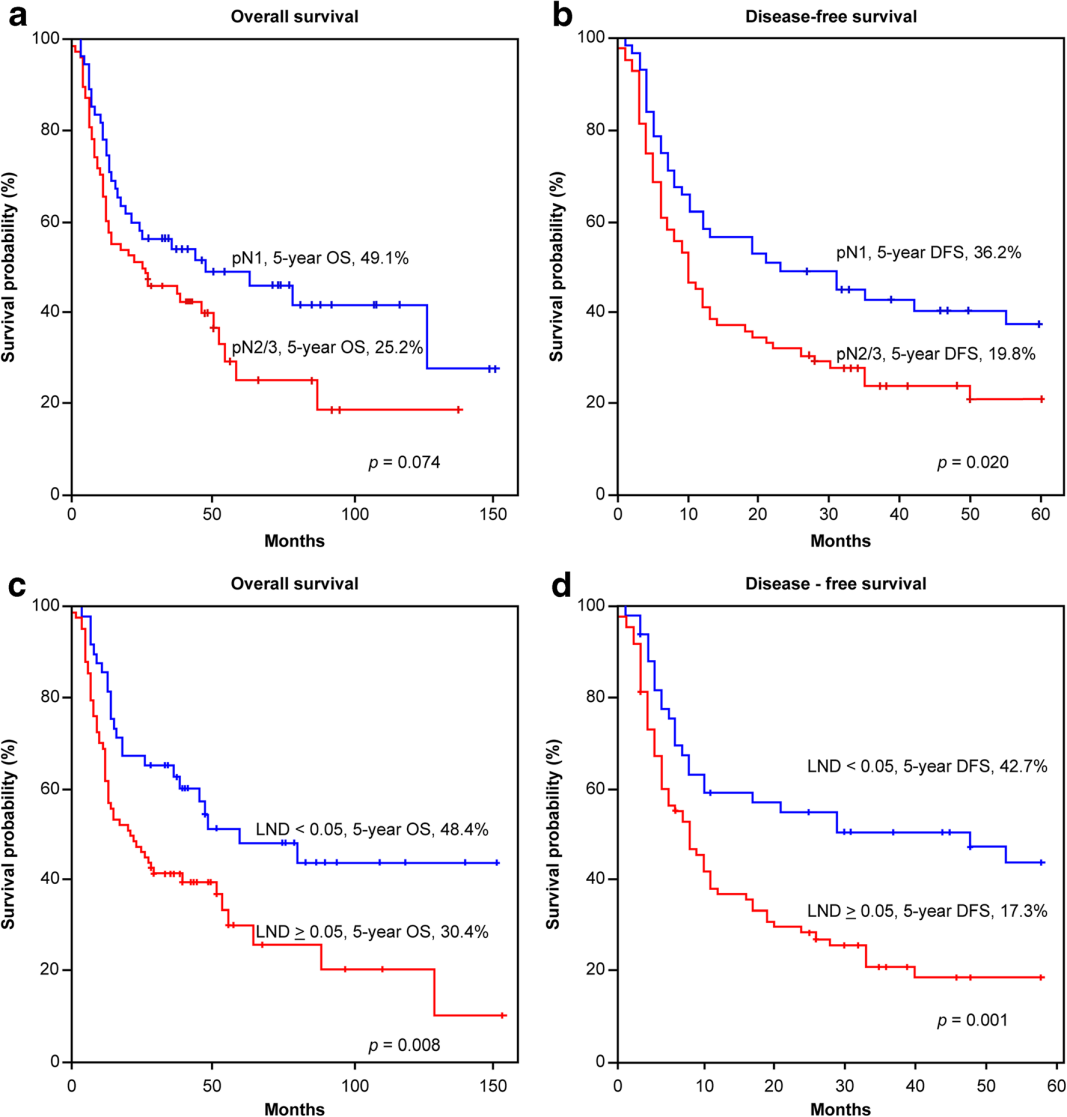
The 5-year overall survival and disease-free survival rates as analyzed by the Kaplan-Meier method in patients with positive histologic lymph nodes: (**a**, **b**) based on tumor-node metastasis (TNM) lymph node classification; (**c**, **d**) based on lymph node density (LND) model with the cutoff point of 0.05

With regard to a positive histological status, the 5-year OS in the pN1 status group and the pN2–3 status group were 49.1 and 25.2%, respectively (*p* < 0.074; Fig. 4a). The 5-year DFS in the pN1 status group and the pN2–3 status group were 46.2 and 19.8%, respectively (*p* = 0.020; Fig. 4b). Based on the LND model with a cutoff point of 0.05 (i.e., LND < 0.05 and LND ≥ 0.05), the 5-year OS was 48.4 and 30.4%, respectively (*p* = 0.008; Fig. 4), and the 5-year DFS was 42.7 and 17.3%, respectively (*p* = 0.001; Fig. 4d).

In reviewing the LND as a prognostic factor, univariate and multivariate models were constructed (Table 3). The variables measured were sex, age, pT classification, pN status, tumor differentiation, stage, and LND. Many factors were significant in the univariate analysis; however, only age, tumor differentiation, and LND were significant predictors of OS (*p* < 0.050). Only tumor differentiation and LND were found to be significant predictors of DFS (*p* < 0.050; Table 4). The Cox proportional hazards model was more discriminatory when LND was included (Table 5).

**TABLE 3:** Univariate and multivariate analysis of prognostic factors for 5-year overall survival

Variables	5-year survival (%)	OS			
		Univariate analysis *p* value	Multivariate analysis *p* value	Adjusted HR	95% CI
Sex		0.525	0.695		
Male	60.3				
Female	68.0				
Age (year)		0.040*	0.025*		
< 65	62.6			1.0	
≥ 65	49.8			1.6	1.1–2.5
T classification		0.000*	0.105		
T1 + T2	72.3				
T3 + T4	41.4				
Differentiation		0.000*	0.002*		
Well	78.5			1.0	
Moderate	59.4			1.4	0.9–2.2
Poor	43.0			2.4	1.4–4.2
N classification		0.000*	0.857		
N0	72.6				
N1	49.1				
N2 + N3	25.2				
Stage		0.000*	0.097		
I	84.2				
II	77.9				
III	54.3				
IV	35.5				
LND		0.000*	0.004*		
0	72.6			1.0	
≤ 0.05	48.4			1.2	0.6–2.4
> 0.05	30.4			2.2	1.3–3.7

*CI*, confidence interval; *HR*, hazard ratio; *LND*, lymph node density; *N*, node; *OS*, overall survival, *T*, tumor

*Indicates a significant difference, *p* < 0.05

**TABLE 4:** Univariate and multivariate analysis of prognostic factors for disease-free survival

Variables	5-year disease-free survival (%)	DFS			
		Univariate analysis *p* value	Multivariate analysis *p* value	Adjusted HR	95% CI
Sex		0.668	0.264		
Men	47.8				
Women	60.6				
Age		0.169	0.139		
< 65	50.2				
≥ 65	40.5				
T classification		0.000*	0.174		
T1 + T2	58.6				
T3 + T4	32.3				
Differentiation		0.000*	0.000*		
Well	61.6			1.0	
Moderate	51.8			1.2	0.8–1.7
Poor	23.9			2.4	1.5–3.9
N classification		0.000*	0.490		
N0	60.1				
N1	36.2				
N2 + N3	19.8				
Stage		0.000*	0.093		
I	70.7				
II	61.9				
III	47.6				
IV	25.1				
LND		0.000*	0.001*		
0	60.1			1.0	
≤ 0.05	42.7			1.1	0.6–2.2
> 0.05	17.3			2.3	1.4–3.7

*CI*, confidence interval; *DFR*, disease-free survival; *HR*, hazard ratio; *LND*, lymph node density; *N*, node; *OS*, overall survival, *T*, tumor

*Indicates a significant difference, *p* < 0.05

**TABLE 5:** Model proportional hazards fit of multivariate analysis

Cox regression model	Without lymph node density	With lymph node density
OS		
-2log likelihood	1628.0	1623.1
*p* value	0.000	0.000*
DFS		
-2log likelihood	1992.6	1985.6
*p* value	0.000	0.000*

*DFS*, disease-free survival; *OS*, overall survival

*Indicates a significant difference, *p* < 0.05

## Discussion

In our study, we used a cutoff point of 0.05 to analyze the OS and DFS via ROC curves and the Youden index (YI), with a higher sensitivity obtained. The LND cutoff value was applied as a predictive factor in our betel nut-chewing patients. However, research regarding the prognosis of patients who chew betel nut is limited, and our LND-based prognosis prediction method is also related to the post operation adjuvant treatment. By using this strategy, we found that a patient’s prognosis was not accurately influenced by TNM staging, since overall survival could not be accurately predicted using the pathologic lymph node status.

The conventional OSCC nodal staging category itself is not adequate for survival analysis and requires a combination of nodal staging categories combined with other factors [15]. Regarding nodal factors, we investigated the LND as a prognostic factor, with the exclusion of cases with positive margins and extracapsular nodal spread. There exists evidence in favor of replacing the conventional TNM staging with other methods, such as pathologic tumor depth [16–19] and cell differentiation [17]. These factors require more comprehensive analyses. In our Cox regression analysis, the conventional TNM staging or specific T and N stages did not demonstrate any significant survival prediction ability; however, LND, tumor differentiation, and age were significant factors for predicting OS (Table 3).

The pathologic nodal stage is critical for adjuvant treatment for oral cancer, and it is based on the specimen’s nodal size and number. In the current study, a mean number of 23 ± 18 lymph nodes (range, 1–84 lymph nodes) were removed in selective neck dissections, and the mean positive nodal metastasis number was 2.8. In previous literature reviews [20, 21], a mean number of 21–50 lymph nodes were removed in a unilateral radical neck dissection, and 1–97 lymph nodes were removed in a unilateral neck dissection [22]. The quantity of lymph nodes removed during dissection in our study was similar to the amounts reported in the literature, but the volume of resected lymph nodes in neck dissection was not included in our previous studies.

The LND applied in analysis was determined to be a more superior predictor of bladder and esophagus carcinoma outcomes, compared to the conventional nodal staging [12, 22]. LND was calculated by the number of positive specimen nodes divided by the total number of dissected nodes. The ratio was postulated as a more useful prognostic factor in survival analysis, in relation to certain treatment characteristics. First, the tumor nodal metastasis ability is determined by the number of histopathologic-positive nodes. The pathological status such as the same stage of pN2b in 2–3 ipsilateral histopathologic-positive nodes did not precisely reflect the tumor migration ability in lymph node metastasis. The migration of the lymph nodes is likewise the numerator of the LND. Second, the treatment characteristic regarding the amount of resected nodal tissue is subject to a surgeon’s preference. The choices for lymph node dissection are selective neck dissection, suprahyoid neck dissection, supraomohyoid neck dissection, radical neck dissection, and modified radical neck dissection. A surgeon’s preference for the amount of nodal tissue removed is the denominator of the LND value. Third, the pathologist’s bias in the sampling procedure is able to relieve the bias via the division to get the LND value.

In our study, the LND cutoff value of 0.05 was used in the OS and DFS analyses of betel nut-chewing habit. For comparison, in 2009, LND was first used for in a survival analysis of OSCC, with a cutoff value of 0.06 [23]. Another study used an LND cutoff of 0.07 for an analysis of OS and DFS in patients with OSCC [24]. Furthermore, one report showed that an LND cutoff value of 0.06 was a significant prognostic factor for OS and DFS in patients with tongue cancer [25]. In another study, an LND value of 0.07 was applied as a predictive factor for lung metastases in OSCC patients [26]. The cutoff values were not similar among these studies. Moreover, the most common etiology of oral cancer was cigarette exposure and alcoholism; betel nut exposure was not considered an etiology. In the population with major betel nut habits and consumption of cigarettes and alcohol, lymph node dissection in the oral regions exposed to betel nut requires the removal of a greater number of lymph nodes or a secondary operation after the pathologic report. In this manner, the final LND value can reach the target of below 0.05.

This study had some limitations. The limited sample size of this study was associated with patient enrollment and poor follow-up compliance. Therefore, future studies should utilize a larger number of patients. In addition to LND, the pathologic pattern of lymphatic metastases features, such as size, volume, and extracapsular spread, should be analyzed as prognostic factors in future studies. Although LND could potentially be a useful prognostic tool, there appears to be several issues that should be addressed, such as surgeon’s choice for the type of neck dissection (radical, selective, and functional), as well as surgeon’s skill in removing all available nodes. A simple LND value may need to be further stratify which type of neck dissection should be performed and which levels (such as level I to VI) of the neck are included, with each site investigated to determine their own designated LND cutoff. Since the quantity of nodes present at each level of the neck is not necessarily proportional in terms of tissue drainage along the superior-inferior direction, LND values could be affected. We are planning a future study with more specification in regard to the lymphatic sites and surgical dissection types, as well as investigation into the specific LND types for each location and surgeon’s preference. Furthermore, addressing these limitations in future studies may establish a more comprehensive prognosis evaluation system.

## Conclusions

Lymph node density was validated as a significant predictor in the OS and DFS analysis, and an LND value of 0.05 generated more accurate survival analyses in patients with betel nut exposure. Based on the predicator, further adjuvant treatment such as reoperation or adjuvant radiotherapy should be indicated for betel nut-chewing patients.

## References

[1] Lo WL, Kao SY, Chi LY, Wong YK, Chang RC (2003). Outcomes of oral squamous cell carcinoma in Taiwan after surgical therapy: factors affecting survival. J Oral Maxillofac Surg.

[2] Lee CH, Ko AM, Yen CF (2012). Betel-quid dependence and oral potentially malignant disorders in six Asian countries. Br J Psychiatry.

[3] Kao SY, Lim E (2015). An overview of detection and screening of oral cancer in Taiwan. Chin J Dent Res.

[4] IARC Working Group on the Evaluation of Carcinogenic Risks to Humans (2004). Betel-quid and areca-nut chewing and some areca-nut derived nitrosamines. IARC Monogr Eval Carcinog Risks Hum.

[5] Head and Neck Cancers (Version 2.2016). (Accessed October 16, 2016, at https://www.nccn.org/professionals/physician_gls/pdf/head-and-neck.pdf)

[6] Lyons AJ, Jones J (2007). Cell adhesion molecules, the extracellular matrix and oral squamous carcinoma. Int J Oral Maxillofac Surg.

[7] Daley T, Darling M (2003). Nonsquamous cell malignant tumours of the oral cavity: an overview. J Can Dent Assoc.

[8] Jemal A, Siegel R, Ward E, Hao Y, Xu J (2009) Thun MJ. Cancer statistics, 2009. CA Cancer J Clin 59:225–249. doi: 10.3322/caac.20006 10.3322/caac.20006 19474385

[9] Yanamoto S, Otsuru M, Ota Y (2015). Multicenter retrospective study of adjuvant therapy for patients with pathologically lymph node-positive oral squamous cell carcinoma: analysis of covariance using propensity score. Ann Surg Oncol.

[10] Kreppel M, Nazarli P, Grandoch A (2016). Clinical and histopathological staging in oral squamous cell carcinoma—comparison of the prognostic significance. Oral Oncol.

[11] Stein JP, Cai J, Groshen S, Skinner DG (2003). Risk factors for patients with pelvic lymph node metastases following radical cystectomy with en bloc pelvic lymphadenectomy: concept of lymph node density. J Urol.

[12] Ooki A, Yamashita K, Kobayashi N (2007). Lymph node metastasis density and growth pattern as independent prognostic factors in advanced esophageal squamous cell carcinoma. World J Surg.

[13] Guo SE, Huang TJ, Huang JC (2013). Alcohol, betel-nut and cigarette consumption are negatively associated with health promoting behaviors in Taiwan: a cross-sectional study. BMC Public Health.

[14] Youden W (1950). Index for rating diagnostic tests. Cancer.

[15] Ebrahimi A, Gil Z, Amit M (2016). International Consortium for Outcome Research in Head and Neck Cancer. Comparison of the American Joint Committee on cancer N1 versus N2a nodal categories for predicting survival and recurrence in patients with oral cancer: time to acknowledge an arbitrary distinction and modify the system. Head Neck.

[16] Abu-Serriah M, Shah KA, Rajamanohara R et al (2015) Tumour depth of invasion of pT1 squamous cell carcinoma of the oral tongue and risk of pathologically detected neck metastases. Head Neck. 10.1002/hed.24139 10.1002/hed.24139 26040258

[17] Haksever M, Inançli HM, Tunçel U (2012). The effects of tumor size, degree of differentiation, and depth of invasion on the risk of neck node metastasis in squamous cell carcinoma of the oral cavity. Ear Nose Throat J.

[18] Pentenero M, Gandolfo S, Carrozzo M (2005). Importance of tumor thickness and depth of invasion in nodal involvement and prognosis of oral squamous cell carcinoma: a review of the literature. Head Neck.

[19] Tan WJ, Chia CS, Tan HK, Soo KC, Iyer NG (2012). Prognostic significance of invasion depth in oral tongue squamous cell carcinoma. ORL J Otorhinolaryngol Relat Spec.

[20] Bhattacharyya N (1998). The effects of more conservative neck dissections and radiotherapy on nodal yields from the neck. Arch Otolaryngol Head Neck Surg.

[21] Jose J, Coatesworth AP, MacLennan K (2001). Cervical metastases in upper aerodigestive tract squamous cell carcinoma: histopathologic analysis and reporting. Head Neck.

[22] Agrama MT, Reiter D, Topham AK, Keane WM (2001). Node counts in neck dissection: are they useful in outcomes research?. Otolaryngol Head Neck Surg.

[23] Gil Z, Carlson DL, Boyle JO (2009). Lymph node density is a significant predictor of outcome in patients with oral cancer. Cancer.

[24] Patel SG, Amit M, Yen TC (2013). International Consortium for Outcome Research (ICOR) in Head and Neck Cancer. Lymph node density in oral cavity cancer: results of the International Consortium for Outcomes Research. Br J Cancer.

[25] Ong W, Zhao R, Lui B (2016). Prognostic significance of lymph node density in squamous cell carcinoma of the tongue. Head Neck.

[26] Suzuki H, Beppu S, Hanai N, Hirakawa H, Hasegawa Y (2016). Lymph node density predicts lung metastases in oral squamous cell carcinoma. Br J Oral Maxillofac Surg.

